# Small Molecule Inhibition of *Mononegavirales* RNA Polymerases

**DOI:** 10.1002/jmv.70972

**Published:** 2026-05-18

**Authors:** Claire R. Cao, Meer Mohammed, Ge Yang, Dong Wang

**Affiliations:** ^1^ Section of Transcription & Gene Regulation The Hormel Institute, University of Minnesota Austin Minnesota USA; ^2^ The Blake School Minneapolis Minnesota USA; ^3^ Department of Biochemistry & Chemistry Wartburg College Waverly Iowa USA

**Keywords:** cryo‐EM, mononegavirales, non‐nucleoside inhibitor, nucleoside and nucleotide inhibitor, RNA polymerase

## Abstract

The order *Mononegavirale*s includes several non‐segmented negative‐sense RNA viruses that are significant human pathogens, whose transcription and replication depend on multifunctional RNA‐dependent RNA polymerase (L protein). Recent discoveries in cryo‐electron microscopy (cryo‐EM) have elucidated the structures of these polymerases and their interactions with small‐molecule inhibitors, providing possible insights for antiviral design. This review summarizes current developments and discoveries in small‐molecule inhibition of *Mononegavirales* polymerases, exploring nucleoside/nucleotide inhibitors that target the catalytic site and non‐nucleoside inhibitors that engage allosteric pockets within the L protein. By combining biochemical and structural findings, these studies reveal conserved druggable pockets, providing opportunities for the development of broad‐spectrum antivirals drugs against *Mononegavirales* pathogens.

## 1 Introduction

**Figure 1:**
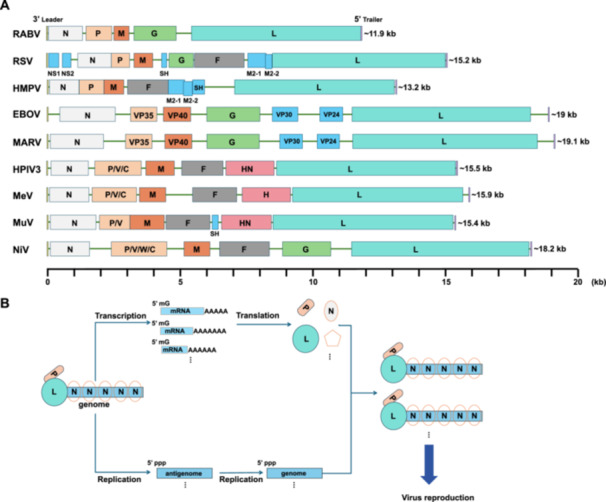
The genome organization and RNA synthesis of mononegaviruses. (A) Representative genomes of human‐pathogenic mononegaviruses are depicted from the 3′ end to the 5′ end. The 3′ leader region is shown in yellow, and the 5′ trailer region is shown in purple. The ORFs encoding viral structural proteins are colored as follows: nucleoprotein (N, light gray), phosphoprotein (P or VP35, light orange), matrix protein (M or VP40, orange), glycoprotein (G, light green), fusion protein (F, dark gray), hemagglutinin or hemagglutinin‐neuraminidase (H or HN, pink), and the polymerase (L, light blue). The ORFs encoding viral non‐structural proteins, including NS1, NS2, SH, M2‐1, M2‐2, VP30 and VP24 are shown in blue. Additional P‐cistron encoded proteins such as V, W, and C are indicated within the P ORF box. The complete viral genome sequences from GenBank are as follows: RABV: NC_001542.1; RSV: FJ614813.1; HMPV: PV218189.1; EBOV: MG572235.1; MARV: NC_001608.4; HPIV3: NC_001796.2; MeV: NC_001498.1; MuV: JN012242.1; NiV: NC_002728.1. (B) Schematic diagrams illustrating how RNA polymerase catalyzes mRNA transcription and genome replication, thus facilitating nucleocapsid formation and subsequent mononegavirus reproduction.

*Mononegavirales* is an order of non‐segmented, negative‐strand RNA viruses (nsNSVs) [1, 2]. It includes highly pathogenic viruses responsible for severe human diseases, such as members of *Rhabdoviridae* including rabies virus (RABV) [3], *Pneumoviridae* including human respiratory syncytial virus (RSV) [4] and human metapneumovirus (HMPV) [5], *Filoviridae* including Ebola virus (EBOV) [6, 7] and Marburg virus (MARV) [7], and *Paramyxoviridae* including human parainfluenza virus 3 (HPIV3) [8], measles virus (MeV) [9], mumps virus (MuV) [10], and Nipah virus (NiV) [11, 12]. The genome organization is linear and single‐stranded, with their lengths ranging from 11.9 to 19.1 kilobases and encoding between 5 and 11 genes (Figure 1A). Three conserved proteins, the nucleoprotein (N), which is responsible for RNA encapsidation, the phosphoprotein (P or VP35), which functions as an RNA polymerase cofactor, and the large protein (L), which serves as the RNA‐dependent RNA polymerase, assemble into the ribonucleoprotein complex (RNP). This core RNA synthesis machinery is essential for both transcription and replication of the viral genome (Figure 1B). Within this complex, the L protein serves as the central catalytic component and therefore critical for the life cycle of *Mononegavirales*.

In antiviral research, the discovery and development of small molecules that target viral polymerases has long been a major focus. Based on how they inhibit viral replication, these compounds are broadly classified into either nucleoside/nucleotide inhibitors (NIs) or non‐nucleoside inhibitors (NNIs). Recent studies have identified a growing number of drug candidates capable of targeting human mononegaviruses. In addition, advancements in cryo‐electron microscopy (cryo‐EM) have allowed for the direct visualization of inhibitors and their interactions with mononegaviral RNA polymerases. Table 1 summarizes representative inhibitors targeting *Mononegavirales* polymerases, including their target viruses, available structural data, and clinical development stages. These discoveries help validate the binding modes of candidate compounds in addition to revealing the mechanisms in which small‐molecule inhibition is conserved across various viral families. Here, we provide an overview of these polymerase‐targeting molecules, investigating biochemical and structural insights to determine their therapeutic potential and efficacy against human mononegaviruses.

**Table 1:** . Representative nucleoside/nucleotide inhibitors (NIs) and non‐nucleoside inhibitors (NNIs) targeting Mononegavirales polymerases.

*Mononegavirales*		NIs		NNIs		
Family	virus	Drug	Clinical stage	Drug	PDB code	Clinical stage
*Rhabdoviridae*	rabies virus (RABV)	Ribavirin	Preclinical	n/a		
		Favipiravir	Preclinical			
		Molnupiravir	Preclinical			
*Pneumoviridae*	human respiratory syncytial virus (RSV)	Ribavirin	Approved	MRK‐1	8FPI	Preclinical
				JNJ‐8003	9ECV, 9ED2, 8FU3	In vitro
		VV116(Remdesivir)	II			
		Obeldesivir	II	JNJ‐2729	9C7Y	In vitro
				Compound 22	9N36	In vitro
	Human metapneumovirus (HMPV)	Ribavirin	Off‐label use	MRK‐1	8FPJ	In vitro
		Favipiravir	Preclinical			
*Filoviridae*	Ebola virus (EBOV)	Remdesivir	III	Suramin	7YET	In vitro
		Favipiravir	II			
	Marburg virus (MARV)	Remdesivir	Preclinical	n/a		
		Favipiravir	Preclinical			
*Paramyxoviridae*	human parainfluenza viruse 3 (HPIV3)	Ribavirin	Off‐label use	n/a		
		GS‐441524 (Remdesivir)	Preclinical			
	Measles virus (MeV)	Ribavirin	Off‐label use	AS‐136A	9KNV	In vitro
		Favipiravir	In vitro	ERDRP‐0519	9OCE, 9OCF, 9KNT	Preclinical
		Remdesivir	In vitro			
	Mumps virus (MuV)	Ribavirin	In vitro	n/a		
		Favipiravir	In vitro			
	Nipah virus (NiV)	Ribavirin	Clinical use (in outbreaks)	ERDRP‐0519	9KNZ	In vitro
		Remdesivir	Preclinical			
		Favipiravir	Preclinical			

*Note:* For targeting *Mononegavirales* polymerase, the NIs listed here are highly selective and not comprehensive of all inhibitors, and only NNIs with published polymerase‐bound structures are summarized, with their PDB codes included.

Abbreviations: NIs, nucleoside/nucleotide inhibitors; NNIs, non‐nucleoside inhibitors.

### 1.1 The RNA Polymerase

The negative‐sense RNA genome of mononegaviruses serves as the template for both transcription and replication [13, 14]. During transcription, the L protein initiates de novo RNA synthesis by recognizing the promoter within the leader (Le) region at the 3′ end of the genome [15]. The L protein first generates the Le RNA transcript, which remains uncapped and non‐polyadenylated. Once synthesized, the Le RNA is released, after which the L protein continues along the template to transcribe downstream genes. At each gene junction, transcription is regulated by cis‐acting signals, the L protein initiates and caps mRNAs at gene‐start (GS) sequences and terminates and polyadenylates them at gene‐end (GE) sequences [16, 17, 18]. In contrast, during replication, the L protein also initiates at the Le region but disregards these cis‐acting regulatory elements, thus generating a full‐length uncapped antigenome. This antigenome then serves as the template for complementary synthesis of negative‐sense genome, initiated by the L protein at the 3′ end of the trailer (Tr) region [19].

**Figure 2:**
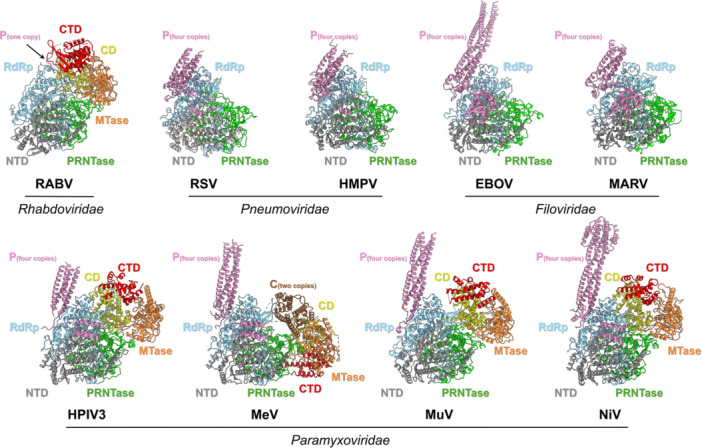
Domain organization and overall structure of *Mononegavirales* polymerases. RNA polymerases from representative virus families in *Mononegavirales* are shown as L proteins in complex with their corresponding cofactors (P, VP35, or C proteins). The domain architecture of the L protein is divided into six parts: the N‐terminal domain (NTD, gray), RNA‐dependent RNA polymerase (RdRp, light blue), polyribonucleotidyltransferase (PRNTase, green), connector domain (CD, yellow), methyltransferase (MTase, orange), and C‐terminal domain (CTD, red). The P protein is shown in pink, and the C protein is shown in brown. For RSV, HMPV, EBOV, and MARV, only the NTD‐RdRp‐PRNTase regions are resolved in the available structures. RABV L is shown bound to a single P protomer, whereas the other structures show L bound to tetrameric P. MeV L is additionally shown in complex with dimeric C proteins. The models used here in PDB code are RABV (6UEB), RSV (6PZK), HMPV (6U5O), EBOV (7YER), MARV (9IP2), HPIV3 (8KDC), MeV (9DUT), MuV (8IZL), NiV (9GJU).

**Figure 3:**
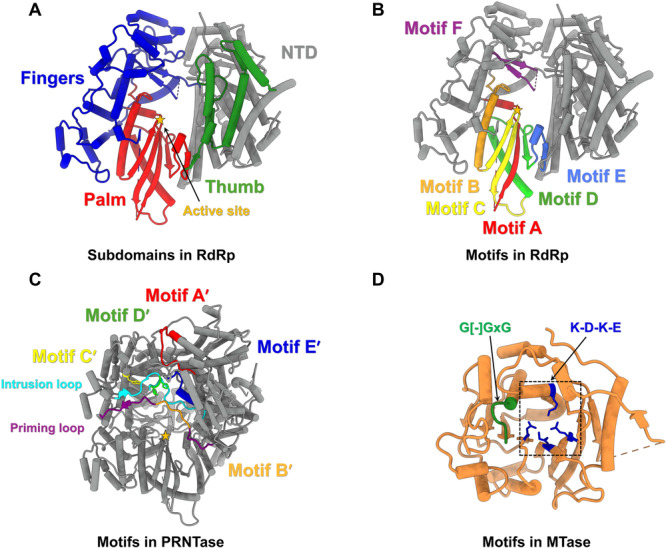
The conserved catalytic cores of the RSV polymerase. (A) The RdRp domain contains three subdomains: fingers (residues 433–689 and 709–772, blue), palm (residues 690–708 and 773–877, red), and thumb (residues 878–968, green). The active site is indicated by an orange asterisk. (B) The conserved motifs (A–F) in the RdRp domain are as follows: motif A (residues 690–708, red), motif B (residues 773‐801, orange), motif C (residues 802–827, yellow), motif D (residues 828–865, lime), motif E (residues 866–876, royal blue), and motif F (residues 613–632, purple). (C) The conserved motifs (A′–E′) in PRNTase domain are as follows: motif A′ (residues 1207‐1222, red), motif B′ (residues 1262‐1267, orange), motif C′ (residues 1297, yellow), motif D′ (residues 1338–1339, lime), and motif E′ (residues 1380–1389, medium blue). Two loops, including priming loop (residues 1256‐1276, purple) and intrusion loop (residues 1329–1353, cyan), are critical for PRNTase activity. (D) The MTase domain contains the conserved catalytic K‐D‐K‐E motif (K1673‐D1779‐K1817‐E1848), which is common to 2′‐O methyltransferases, and the G[‐]GxG motif (1696‐GEGAG‐1700) forms part of the SAM binding site. The RSV RdRp and PRNTase domains are shown using PDB ID 6PZK, and the MTase domain is shown using PDB ID 4UCI.

The L protein is a multidomain enzyme that carries out the entire processes of transcription and replication, conducting all catalytic activities required for RNA synthesis (Figures 2, 3). These domains include the RNA‐dependent RNA polymerase (RdRp), the polyribonucleotidyltransferase (PRNTase), and the methyltransferase (MTase) domains [13, 20, 21]. The RdRp domain is responsible for catalyzing nucleotide polymerization, which synthesizes RNA. The PRNTase domain is responsible for capping nascent viral mRNAs and the MTase domain is responsible for carrying out sequential methylation of the RNA cap structure to stabilize RNA and ensure efficient translation. Additional structural modules, such as the N‐terminal domain (NTD), connector domain (CD), and C‐terminal domain (CTD), help coordinate these catalytic activities. In addition, the P protein serves as a critical cofactor that stabilizes the L protein and binds to the N protein, ensuring the L protein remains in the proper location of the nucleocapsid, therefore facilitating polymerase function within the host cell [20, 21, 22].

The RdRp domain adopts a right‐hand architecture composed of fingers, palm, and thumb subdomains (Figure 3A) [13], and contains the conserved structural motifs A–F (Figure 3B) [23]. Motif A contains a conserved aspartic acid residue that binds to metal ions such as magnesium, which is crucial for the enzymatic reaction [24]. Motif B is involved in NTP‐ribose selection, template binding, and RNA translocation [25]. Motif C contains the GDN sequence that is critical for catalysis [13]. Motif D forms a strand‐turn‐helix and Motif E forms a subsequent β‐hairpin structure, both of which may serve to support the architecture of the palm subdomain. Motif D has been suggested to contribute to the fidelity of nucleotide incorporation [26], whereas Motif E may participate in positioning the 3′ end of the elongating RNA transcript [27]. Motif F, found in the fingers subdomain, interacts with the triphosphate group of the incoming NTP [28], which is essential for transcription and replication [29].

The PRNTase domain contains five highly conserved amino acid sequence elements (Figure 3C), Rx(3)Wx(3‐8)ΦxGxζx(P/A) (motif A′; Φ, hydrophobic, ζ, hydrophilic, x, any amino acid), (Y/W)ΦGSxT (motif B′), W (motif C′), HR (motif D′), and ζxxΦx(F/Y)QxxΦ (motif E′) [30]. Motifs B′–E′ are located close to the center, forming the active site of the enzyme, while a helix‐loop structure that contains motif A′ likely plays a structural role by providing a platform for organization of the PRNTase active site [23]. Two critical loops, the priming loop and the intrusion loop, are located near the RdRp active site (Figure 3C). The priming loop is essential for the initiation of RNA synthesis [31, 32]. The intrusion loop is required for PRNTase activity during formation of the enzyme‐pRNA intermediate [33]. During the capping of the nascent RNA strand, the first nucleotide forms a covalent bond with the histidine residue in the HR motif D′ of the intrusion loop. Cap addition then proceeds through a nucleophilic attack by GDP, and the GxxT motif B′ within the priming loop likely facilitates binding of the capping guanosine [34].

The MTase domain, located between the CD and CTD domains, adopts a typical S‐adenosyl‐l‐methionine (SAM)‐dependent methyltransferase fold (Figure 3D). The domain also features a glycine‐rich SAM‐binding motif (G[‐]GxG), along with a catalytic K‐D‐K‐E motif characteristic of 2′‐O‐methyltransferases [34, 35].

Given the L protein requires such complicated coordination of multiple catalytic centers, viral replication can be effectively derailed via the disruption of any of these enzymatic steps. Inhibition of the RdRp active site can directly prevent nucleotide incorporation and elongation of the RNA. Additionally, interference with the PRNTase or MTase domains can prevent mRNA capping and methylation, respectively. Such direct or allosteric inhibition of catalytic activity by small molecules thus creates a potential pathway for antiviral development.

### 1.2 Nucleoside and Nucleotide Inhibitors

Nucleoside and nucleotide inhibitors (NIs) are small molecules that mimic natural nucleoside triphosphates (NTPs) and compete for binding at the polymerase catalytic site [36]. In order to become active, NIs require intracellular bioactivation through phosphorylation. Nucleoside analogs enter cells in a non‐phosphorylated form and undergo three sequential phosphorylation steps. On the other hand, nucleotide analogs enter as mono‐phosphorylated derivatives and require only two additional steps to reach the active triphosphate form. Once converted, these active metabolites are incorporated into the growing RNA chain, where they act either as obligate or non‐obligate chain terminators. In doing so, they disrupt normal RNA synthesis and effectively stop genome replication and transcription. Recent discoveries have greatly expanded the arsenal of nucleoside and nucleotide inhibitors across multiple viral families. However, their effectiveness often varies among different viruses since some compounds remain limited by metabolic activation, selectivity, or toxicity. The following sections review the representative NIs characterized in various viruses, dissecting their mechanisms as well as their potential in preventing replication.

### 1.3 Rhabdoviridae

RABV causes a vaccine‐preventable infectious disease that targets the central nervous system (CNS) of both animals and humans. The guanine nucleoside analog ribavirin has been shown to have effective inhibitory effects on RABV replication in cell culture [37]. When directly incorporated into viral RNA, ribavirin induces lethal mutagenesis during genome replication. Furthermore, ribavirin also inhibits inosine monophosphate dehydrogenase (IMPDH), therefore depleting the intracellular GTP pool required for viral RNA synthesis [37]. Favipiravir, a pyrazine‐derived broad‐spectrum RNA polymerase inhibitor, has likewise shown antiviral activity against RABV in vitro [38]. However, both ribavirin and favipiravir fail to achieve sufficient CNS concentrations in vivo to be effective against rabies [39, 40]. Molnupiravir, a prodrug of N‐hydroxycytidine (NHC), exhibits comparable in vitro anti‐RABV activity to ribavirin and favipiravir. However, prophylactic administration of molnupiravir in RABV‐infected mice did not result in improved survival or reduced viral titers in the brain [41]. It should be noted that, compared with many other RNA viruses, rhabdovirus antivirals must overcome the additional challenge of blood‐brain barrier penetration to treat neurotropic infections such as rabies.

### 1.4 Pneumoviridae

RSV causes severe lower respiratory tract infections (LRIs) and is a major cause of morbidity and mortality in high‐risk populations [42]. Ribavirin has been tested for its efficacy against RSV both in vitro [43] and in vivo [44], and has been approved for use in hospitalized infants and young children with severe LRIs. However, its clinical use remains limited due to its uncertain efficacy and complexity in administration [45]. The oral remdesivir derivative VV116 has also been reported to act as a potent inhibitor of RSV replication, showing efficacy both in vitro and in mouse models [46]. Furthermore, oral administration of obeldesivir has been tested as effective against RSV infection in African green monkeys [47], making it a promising drug for future clinical evaluation.

HMPV is another major cause of respiratory tract infections [48]. The development of NIs for HMPV is largely similar to RSV, as both of these viruses belong to the *Pneumoviridae* family. Ribavirin has shown comparable in vitro activity against HMPV and RSV [49], and these findings have been further supported by in vivo studies in BALB/c mice [50]. However, there still lacks a randomized controlled trial testing for ribavirin treatment for HMPV infection. Furthermore, safer and more effective antiviral agents are needed due to concerns regarding its teratogenicity and high production [51]. Favipiravir has also demonstrated antiviral potency, effectively inhibiting HMPV replication in vitro and in vivo when administered early during infection, although additional studies are needed before clinical evaluation [52].

### 1.5 Filoviridae

EBOV is a highly virulent pathogen that causes severe hemorrhagic fever with a case fatality rate ranging from 50% to 90% [53]. Remdesivir competes with adenosine triphosphate (ATP) for incorporation by the viral RNA polymerase and acts as a non‐obligate delayed chain terminator [54]. Its therapeutic efficacy has also been evaluated in vivo studies using rhesus monkey models of Ebola virus infection [55]. During the PALM clinical trial conducted amid the Ebola virus epidemic, remdesivir was evaluated alongside the monoclonal antibody therapies ZMapp, mAb114, and REGN‐EB3. However, mAb114 and REGN‐EB3 were determined to be greater efficacy compared to remdesivir [56, 57]. Favipiravir conferred 100% protection when orally administered to immunodeficient mice against aerosol Ebola virus E718 infection [58]. However, during the JIKI clinical trial conducted in Guinea, favipiravir failed to achieve the targeted plasma concentrations in patients infected with EBOV, highlighting the need to establish safe and effective dosing ranges in healthy volunteers prior to efficacy studies [59]. These findings highlight the need for continued evaluation and optimization of NI candidates, as well as the development of more effective polymerase inhibitors against EBOV.

MARV causes a highly virulent hemorrhagic fever with a case fatality rate of up to 88% [60]. Remdesivir combined with monoclonal antibody treatment has been shown to extend the therapeutic window and provide significant protection in a non‐human primate model of MARV disease, especially when initiated at a critical point in disease progression [61]. Furthermore, promising results were obtained when intravenous favipiravir was administered to six cynomolgus macaques infected with MARV, resulting in 83% survival (five of six animals), whereas all untreated controls died, making it a promising drug for future clinical trials [62].

### 1.6 Paramyxoviridae

HPIVs are responsible for causing common acute upper and lower respiratory infections across all age groups, with especially young children being the most vulnerable [63]. Among the four recognized HPIV serotypes, HPIV3 is most frequently associated with severe lower respiratory diseases, particularly in children under 5 years of age and in older adults [64]. In a retrospective analysis of allogeneic stem cell transplantation recipients with human parainfluenza virus infection, ribavirin treatment was associated with a significantly lower risk of progression from upper to lower respiratory tract HPIV3‐induced disease [65]. In a newly developed AG129 mouse model of HPIV‐3 infection, oral administration of GS‐441524, the parent nucleoside of remdesivir, significantly reduced infectious virus titers in the lung and preserved near‐normal lung histology [66]. Notably, the combination of ribavirin and GS‐441524 or remdesivir was found to be effective against HPIV3 infection in human airway epithelial cell cultures and in a mouse infection model [67].

MeV causes a highly contagious respiratory illness that continues to pose significant global and national public health challenges despite progress in vaccination efforts [68]. Although measles was declared eliminated in 2000, there has been a rise in cases in recent years, particularly within populations with low vaccination coverage [69]. Ribavirin demonstrates in vitro activity against measles virus [70]. While ribavirin has been administered to patients with severe measles disease [71, 72], clinical evidence supporting its efficacy is limited. In addition, favipiravir [73] and remdesivir [74] have demonstrated antiviral activity against the measles virus in vitro.

MuV typically causes fever and swelling of the parotid salivary glands. Although widespread vaccination has significantly reduced its prevalence, in recent decades, large outbreaks have continued to occur globally even among vaccinated populations [75]. Ribavirin induces lethal mutagenesis in MuV in vitro by increasing genomic diversity. Notably, at higher concentrations, ribavirin completely stops viral infectivity during serial passaging. On the other hand, prolonged exposure at lower concentrations increases MuV population heterogeneity without making the virus resistant to the drug [76]. Favipiravir has also been reported to effectively inhibit the growth of wild‐type and vaccine strains of MuV in vitro at low micromolar concentrations [77].

NiV is an emerging highly pathogenic bat‐borne zoonotic virus. NiV infection can result in severe acute respiratory illness and various forms of encephalitis, including acute, relapsed, and late‐onset disease. Fatality rates range from 40% to 100% [78, 79]. Ribavirin has been used clinically during NiV outbreaks, including the 1998‐1999 Malaysia [80] and 2018 Kerala epidemics [81], with reported reductions in mortality of approximately 36% and 20%, respectively. Although treatment was associated with improved survival rates in some cases, the overall efficacy of ribavirin against NiV is still inconclusive due to limited sample sizes and inconsistent clinical results. Additionally, ribavirin administered either alone [82] or in combination with chloroquine [83] was ineffective at preventing disease progression in the hamster model. However, in a lethal challenge model using African green monkeys infected with the NiV Bangladesh genotype, remdesivir treatment resulted in 100% survival when the drug was administered intravenously once daily, starting 24 h post‐infection and continuing for 12 days [84]. In the Syrian hamster model, favipiravir was effective in preventing lethal NiV infection when administered either orally twice daily or subcutaneously once daily for 14 days [85].

**Figure 4:**
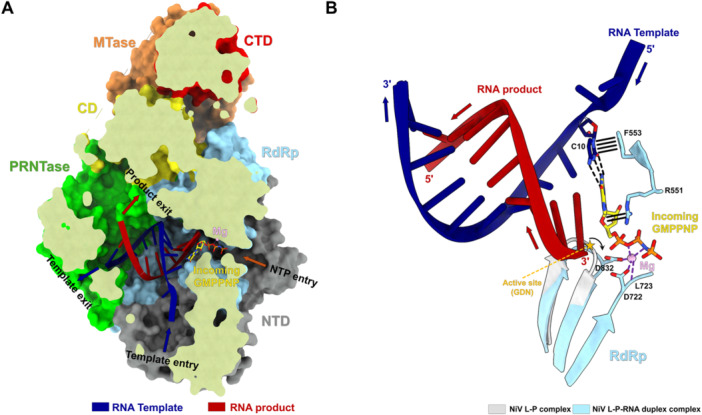
The binding mode of the GTP analogue GMPPNP in the early elongating NiV L‐P complex. (A) Slice‐through view of the early elongating NiV L–P complex with an incoming GMPPNP. The structure is shown as a surface representation with a partially clipped surface. The RNA template and GMPPNP are shown as cartoons and stick, respectively. The template entrance and exit, the NTP entrance and product exit are highlighted. (B) Schematic of key residues involved in GMPPNP binding and the associated active‐site conformational changes in the early elongation NiV L–P complex. The quadruple solid line indicates a π–π interaction between C10 of the RNA template and F553. The double solid line indicates a π–cation interaction between the ribose of GMPPNP and R551. The triple dashed line indicates base‐pair formation between GMPPNP and C10. The purple dashed line indicates magnesium coordination. Superposition of the NiV L–P–RNA complex with the apo NiV L–P complex reveals movement of the active site in the elongation state, indicated by the black arrow. The models used here correspond to NiV L–P (PDB: 9GJT) and NiV L–P–RNA (PDB: 9GJU).

Although no structure of a NI bound to an RNA polymerase from nsNSVs has yet been reported, recent structural studies of the NiV polymerase provide important insights into nucleotide recognition at the active site [12] (Figure 4). An elongation‐state structure of the NiV L‐P polymerase complex with the incoming nucleotide analogue GMPPNP captures substrate binding prior to phosphodiester bond formation (Figure 4A). In this early elongation state, the templating base C10 is stabilized in the +1 position through stacking with the conserved residue F553. The incoming GMPPNP forms canonical base pairing with C10 and is further stabilized by stacking with R551, a residue that protrudes toward the NTP‐binding pocket and is highly conserved among nsNSV polymerases. The triphosphate moiety is coordinated within a catalytic groove formed by motifs A and C, which contain the conserved catalytic aspartates D722 and D832 that orient toward the triphosphate and coordinate a catalytic Mg ion. Comparison of apo and RNA‐bound states further suggests that the active site undergoes conformational rearrangement to accommodate the nascent RNA product (Figure 4B). Although GMPPNP is a non‐hydrolyzable GTP analogue rather than an antiviral compound, this structure provides a snapshot of nucleotide engagement in the polymerase active site and offers a structural framework for understanding how NIs may bind and disrupt RNA synthesis in *Mononegavirales* polymerases.

### 1.7 Non‐Nucleoside Inhibitors

Although no structural information is yet available for NI‐polymerase complexes in *mononegaviruses*, recent cryo‐EM studies have identified multiple NNI‐binding hotspots that allosterically inhibit RNA synthesis.

**Figure 5:**
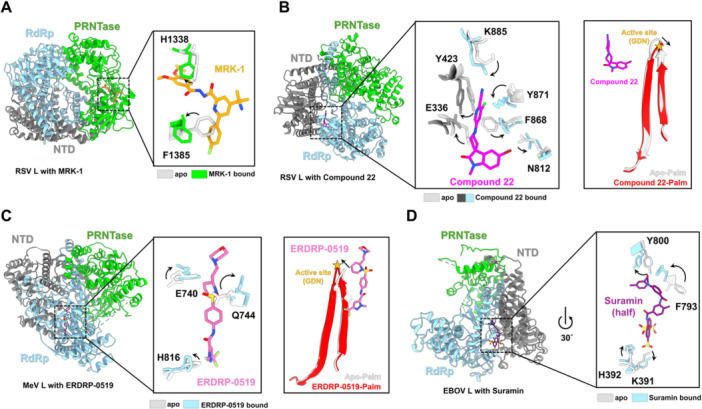
Structural basis and conformational changes induced by NNI binding to *Mononegavirales* polymerases. Structures of the RSV polymerase complex with MRK‐1 (A) and compound 22 (B), the MeV polymerase complex with ERDRP‐0519 (C), and the EBOV polymerase complex with suramin (D). The NNI‐bound polymerase structures are shown on the left in cartoon representation with different subunit coloring. The NNIs (MRK‐1, orange; Compound 22, magenta; ERDRP‐0519, pink; Suramin, purple) are shown as sticks. Zoom‐in: Superimposition of viral RNA polymerases based on the L protein in NNI‐bound and apo states highlights the conformational changes induced upon inhibitor binding. The models used here in PDB code are RSV L‐P‐MRK‐1 (8FPI), RSV L‐P‐compound 22 (9N36), MeV L‐P‐ERDRP‐0519 (9OCF), EBOV L‐P‐suramin (7YET).

MRK‐1 is the first inhibitor whose structure was determined to be in complex with the pneumovirus L protein (Figure 5A) [86]. In RSV, MRK‐1 was observed within a pocket of the PRNTase domain formed by the conserved motifs B′, D′, and E′, with the overall conformation largely unchanged between the apo and MRK‐1‐bound states. However, F1385 undergoes a rotamer rearrangement to accommodate the fluorophenyl group of MRK‐1, forming an edge‐to‐face π interaction, while the side chain of H1338 rotates nearly 90° to fit the inhibitor. The HMPV L protein included a similar, but not identical, MRK‐1 pre‐formed binding pocket. The pre‐formed pocket indicates that instead of relying on an induced‐fit mechanism, MRK‐1 may preferentially bind a low‐energy conformation of the polymerase. Furthermore, functional analyses indicate that MRK‐1 inhibits the conformational changes required for the polymerase to initiate RNA synthesis and transition into elongation. Mutation of the MRK‐1‐interacting residue I1381 was observed to result in drug resistance. Additionally, several related NNIs have been identified in the same PRNTase pocket, including JNJ‐8003 [87, 88] and JNJ‐2729 [89]. Recently, EDP‐323, whose chemical structure has not yet been disclosed but is considered a potential PRNTase‐targeting inhibitor, successfully completed a Phase 2 human challenge study [90].

Compound 22 was recently identified as an NNI that binds independently of previously known allosteric sites in the capping domain of the RSV L‐P polymerase complex (Figure 5B) [91]. Cryo‐EM analysis revealed that compound 22 occupies a novel induced pocket in the palm subdomain of the RdRp, adjacent to motif E, which connects the palm and thumb domains. The inhibitor adopts an l‐shaped conformation and forms multiple stabilizing interactions where the cyano group hydrogen bonds with K885, the pyridine ring engages in π–π stacking with Y423, and the indoline carbonyl oxygen forms a pseudo‐hydrogen bond with G337. The π–π stacking of the indoline ring with Y861 and a halogen bond between the bromo atom and N812 of the conserved GDN motif also provides additional stabilization to the inhibitor binding to the RdRp active site. This halogen bond is essential as substitution of the bromo group with hydrogen (compound 20) resulted in a ~ 10‐fold loss of potency. By inducing conformational rearrangements around motif E and the GDN catalytic motif, compound 22 may sterically hinder the movement of catalytic residues required for phosphodiester bond formation, thus stalling the polymerase during initiation and early elongation.

ERDRP‐0519, a chemically optimized NNI derived from AS‐136A, was observed to have potent and broad‐spectrum activity against morbilliviruses and is currently in late‐lead preclinical development targeting MeV. Recently, the cryo‐EM structures of ERDRP‐0519 bound to the MeV polymerase (Figure 5C) [92, 93] and NiV polymerase [93] have been discovered. It was determined that ERDRP‐0519 inhibits the catalytic center by interacting with residue D773 in the conserved GDN motif. The piperidine group displaces D773, creating space for hydrophobic interactions that suggest a potential impact on active‐site function. Notably, ERDRP‐0519 engages a site on the opposite side of the palm region compared with the binding site of compound 22 (Figure 4B,C). In addition, the morpholine ring may obstruct RNA binding. Cell‐based assays further revealed that a critical hydrogen bond with W671 in MeV contributes to drug resistance [92]. Furthermore, the structure of the NiV L‐P complex in the presence of ERDRP‐0519 reveals a similar binding pocket and suggests conserved inhibition mechanisms [93].

Suramin has been reported as an inhibitor targeting various viruses, including Chikungunya virus [94], norovirus [95], SARS‐CoV‐2 [96], and Ebola virus (EBOV) [6, 94]. In vitro enzymatic assays, with IC₅₀ of about 11 μM and in vivo cell‐based assays with EC₅₀ of about 0.4 μM have shown antiviral activity against EBOV. Suramin is a symmetric polysulfonated naphthylurea. In recent determined EBOV l‐VP35‐suramin complex (Figure 5D) [6], no significant structural changes were observed upon suramin binding. Only several residues, including K391, H392, F793, and Y800, undergo local conformational rearrangements. However, the head half of suramin blocks the NTP entry channel by binding within the RdRp domain. The tail half on the other hand, although with low density, likely extends toward and blocks the nascent RNA exit channel. Furthermore, in vitro and in vivo assays suggest that suramin may also inhibit NiV polymerase [97]. Although the structure of the NiV L‐P‐suramin complex is not yet available, structural superimposition suggests that suramin may act as a broad‐spectrum inhibitor against different nsNSVs by potentially sterically hindering NTP access to the active site.

## 2 Discussion

The growing number of high‐resolution structures of viral RNA polymerases from viruses in the order *Mononegavirales* is reshaping approaches to discovering and developing antiviral solutions. By observing inhibitors within polymerase complexes, structural visualizations provide an accurate basis for drug design and optimization. At the moment, no structure of a NI bound to an RNA polymerase from nsNSVs has been determined. However, the first elongation‐state structure of the NiV polymerase has recently been resolved [12], suggesting that incorporation of NIs into the RNA synthesis process may soon be resolved structurally. In contrast, several NNI‐bound structures of nsNSV polymerases have already been determined, highlighting the different allosteric sites that reveal new potential areas for drug design beyond the typical NTP‐binding pocket targeted by NIs.

Capturing transient intermediates during the polymerase catalytic cycle is essential for determining its functional mechanisms. Recent advances have been made in determining the RNA‐bound structures of nsNSV RNA polymerases. The structures of the EBOV l‐VP35‐RNA complex reveal that the 3′ leader promoter RNA binds within the template entry channel in a distinct, stably bent conformation [98]. In contrast, the overall structures of the RSV polymerase bound to its genomic and antigenomic promoter RNAs are highly similar to that of the apo polymerase [99]. The promoter‐bound states of the EBOV and RSV RNA polymerases, in which the template is positioned toward the catalytic center, likely represent a pre‐initiation state. Furthermore, comparison of the apo and elongation‐state structures of the NiV polymerase shows that the priming loop and intrusion loop become partially ordered upon RNA binding leading to the stabilization of the flexible C‐terminal domains, including the CD, MTase, and CTD [12]. These conformations often involve flexible domain interfaces or partially formed RNA‐protein contacts that can be selectively inhibited by small molecules. Trapping functional states such as pre‐initiation, initiation, elongation, and termination by cryo‐EM could reveal allosteric control points that would otherwise be inaccessible in conventional complexes, allowing for the development of new mechanism‐based inhibitors.

Although the overall architecture of the L protein across *Mononegavirales* is highly conserved, particularly within the NTD‐RdRp‐PRNTase core, inhibitors often show variable efficacy among viral families due to suboptimal pharmacokinetics, limited tissue exposure, inhibitor resistance, and toxicity concerns. While NIs target the conserved RdRp active site, their antiviral potency can still vary across viruses, likely reflecting subtle variations in substrate recognition and active‐site configuration. In contrast, NNIs often bind to allosteric pockets and tend to display virus‐specific activity. For example, MRK‐1 and related compounds inhibit pneumovirus polymerases [86], whereas ERDRP‐0519 has been shown to target MeV [92, 93] and NiV [93], but shows no inhibitory effect on RSV [100]. Compound 22 has so far only been evaluated for its efficacy against RSV [91]. Notably, suramin has been reported to inhibit viruses from multiple families, including EBOV [6] and NiV [97]. Future structural determination of additional inhibitor‐polymerase complexes across different viral families will be important to clarify the molecular basis of these differences and informing future inhibitor optimization.

Looking forward, using combination treatment and targeted protein degradation strategies may increase the efficacy of therapies. Combined inhibitors that engage different polymerase sites could minimize drug resistance. Meanwhile, instead of simply inhibiting essential replication factors, the adaptation of PROTAC (proteolysis targeting chimera) technology to viral proteins [101, 102, 103, 104, 105] could potentially eliminate replication factors such as polymerases entirely. This strategy relies on recruiting host E3 ubiquitin ligases to induce targeted degradation of viral proteins. However, its application to RNA viruses may face challenges, including variable viral protein abundance, intracellular localization, and rapid turnover kinetics. Integrating structural insight with such multi‐mechanistic interventions can potentially develop reliable and broad‐spectrum antiviral therapies capable of stopping future RNA virus outbreaks.

## Author Contributions

Dong Wang and Ge Yang contributed to conceptualization of the manuscript. Claire R Cao and Dong Wang drafted the original manuscript. Claire R Cao, Meer Mohammed and Dong Wang contributed to the visualization of the manuscript. All authors contributed to reviewing and editing of the manuscript. All authors agreed to the final version of the manuscript.

## Conflicts of Interest

The authors declare no conflicts of interest.

## Data Availability

Data sharing not applicable to this article as no datasets were generated or analysed during the current study.
